# Rescue of mutant fitness defects using *in vitro* reconstituted designer transposons in *Mycoplasma mycoides*

**DOI:** 10.3389/fmicb.2014.00369

**Published:** 2014-07-23

**Authors:** Bogumil J. Karas, Kim S. Wise, Lijie Sun, J. Craig Venter, John I. Glass, Clyde A. Hutchison, Hamilton O. Smith, Yo Suzuki

**Affiliations:** ^1^Department of Synthetic Biology and Bioenergy, J. Craig Venter InstituteLa Jolla, CA, USA; ^2^Department of Molecular Microbiology and Immunology, University of MissouriColumbia, MO, USA; ^3^Department of Synthetic Biology and Bioenergy, J. Craig Venter InstituteRockville, MD, USA

**Keywords:** minimal cell, synthetic cell, complementation, transposome, *nusG*

## Abstract

With only hundreds of genes contained within their genomes, mycoplasmas have become model organisms for precise understanding of cellular processes, as well as platform organisms for predictable engineering of microbial functions for mission-critical applications. Despite the availability of “whole genome writing” in *Mycoplasma mycoides*, some traditional methods for genetic engineering are underdeveloped in mycoplasmas. Here we demonstrate two facile transposon-mediated approaches for introducing genes into the synthetic cell based on *M. mycoides*. The marker-less approach involves preparing a fragment containing only a small genomic region of interest with flanking transposase-binding sites, followed by *in vitro* transposase loading and introduction into the cells. The marker-driven approach involves cloning an open reading frame (ORF) of interest into a vector containing a marker for mycoplasma transformation, as well as sites for transposase loading and random genomic integration. An innovative feature of this construct is to use a single promoter to express the transformation marker and the introduced ORF. The marker-driven approach can be conveniently applied to any exogenous or synthetic gene without any information on the effect of the gene on the strain, whereas the marker-less approach requires that the fragment has a recognizable effect. Using the marker-less method, we found that a region containing the *nusG* gene rescues a slow growth phenotype of a strain containing a larger deletion encompassing this gene. Using the marker-driven approach, we better defined this finding, thereby establishing that *nusG* is required for a normal growth rate in synthetic *M. mycoides*. These methods are suitable for complementation tests to identify genes responsible for assorted functions lacking in deletion mutants. These approaches are also expected to facilitate rapid testing of various natural and engineered genes or gene clusters from numerous sources in *M. mycoides*.

## Introduction

The ability to insert, delete, and mutate genes forms the basis of genetic engineering. Organisms with facile tools for these operations have served as model organisms and helped uncover numerous universally applicable principles pertaining to biological processes. However, established model organisms like *Escherichia coli* and *Saccharomyces cerevisiae* have thousands of genes. Although there have been some advances in systematically characterizing these genes (Winzeler et al., 1999; Baba et al., 2006; Hillenmeyer et al., 2008; Costanzo et al., 2010), elucidating how these genes function together as a system to sustain a living organism is not a simple task. In contrast, mycoplasmas have only hundreds of genes in their genomes, yet they are still axenic organisms. As a consequence, the genetic combinatorics needed to understand almost any biological process is much simpler in mycoplasmas than in other microbes. This characteristic makes mycoplasmas uniquely suited for studies aimed at a complete understanding of a cellular system (Smith et al., 2008).

Mycoplasmas are arguably the most advanced bacteria in the field of genomics (Karr et al., 2012; Karas et al., 2013; Maier et al., 2013; Guell et al., 2014). *Mycoplasma genitalium* was one of the first two bacteria to have the whole genome sequenced (Fraser et al., 1995). *M. genitalium* and *Mycoplasma mycoides* were the first two organisms to have the whole genome “written” (Gibson et al., 2008, 2010). In this process, the sequence designed in a computer was used to make synthetic DNA fragments. These fragments were hierarchically assembled to generate a complete genome. The assembled genome of *M. mycoides* was then rebooted in recipient cells of a closely related mycoplasma species to generate a “synthetic cell” (JCVI-syn1.0) controlled solely by the artificial donor genome (Lartigue et al., 2007; Gibson et al., 2010). This method can be used to create almost any sequence within the mycoplasma genome.

The whole genome writing method enables the precise introduction of changes throughout the genome, but because it requires multiple procedures for manipulating large DNA molecules, it is not the most efficient method for introducing a gene or two to evaluate their function in a strain. When challenged with this simple task, mycoplasma research suffers from the shortage of tools (Halbedel and Stulke, 2007). For example, plasmid systems have been developed in only a few selected species (Lartigue et al., 2003; Breton et al., 2012). There have also been only a few expression systems developed so far (Dybvig et al., 2000; Horino et al., 2009; Allam et al., 2010; Chang et al., 2011). Targeted knockout is also inefficient. Therefore, the development of facile tools in mycoplasmas that synergize with the genome synthesis method is expected to greatly accelerate the advance of systems biology research.

**Figure 1:**
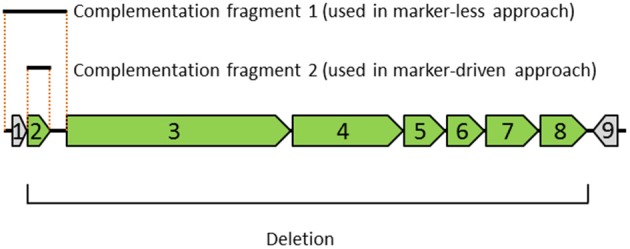
**Diagram depicting the genomic region, deletion and complementation fragments examined in this study**. The genomic region contains nine genes indicated with arrows: (1) MMSYN1_0839, (2) *nusG*, and (3–9) MMSYN1_0841- MMSYN1_0847. The orientation of each gene is indicated (right-pointing arrow denotes a clock-wise direction in the genome). The deletion removes seven genes shown in green (genes 2–8), starting with the initiator codon of gene 2 and ending with the stop codon of gene 8. Complementation fragment 1 includes gene 1, gene 2 and their corresponding upstream and downstream intergenic regions. This complementation fragment was used in a marker-less approach (see Figure 2). Complementation fragment 2 includes only the ORF of gene 2. This fragment was combined with an expression vector and used in a “marker-driven” approach.

One effort in the mycoplasma field is to classify genes into essential genes and non-essential genes using transposon-mediated mutagenesis followed by sequencing (Hutchison et al., 1999; Glass et al., 2006; Hasselbring et al., 2006; French et al., 2008; Mutaqin et al., 2011; Maglennon et al., 2013; Sharma et al., 2014). However, even under saturating conditions, assignment of a gene's essentiality can be ambiguous. Based on one such study in JCVI-syn1.0 (Suzuki et al., in preparation), a deletion of a 7-gene cluster (termed cluster L) containing the genes MMSYN1_0840 (*nusG*), MMSYN1_0841, MMSYN1_0842, MMSYN1_0843, MMSYN1_0844, MMSYN1_0845 and MMSYN1_0846 was generated (Figure 1). This deletion unexpectedly resulted in a slow growth phenotype. To specifically associate this mutant phenotype with one or more of the genes within cluster L, further analysis is needed. Testing single-gene knockouts is a possibility, but potential operon arrangement among the genes may confound such an attempt, requiring elaborate reorganization of sequences to avoid the perturbation of neighboring genes. Because designing reorganized sequences is not always straightforward, a few variants of a genomic construct may need to be tested for each knockout. If a single-gene knockout does not reproduce the phonotype resulting from the cluster deletion, combinations of deletions may need to be tested. In this case, the number of constructs to be tested could become as high as 128 (= 2^7^). Genome synthesis or engineering via DNA assembly in yeast can be used to incorporate these changes, but the associated processes for handling large DNA fragments can be prohibitively labor-intensive. Therefore, when numerous changes confined to a specific region or a small set of genes are tested, direct engineering within mycoplasmas may be more efficient.

In this paper, we demonstrate two simple approaches for introducing genes into synthetic *M. mycoides* using a system for reconstituting active transposon complexes *in vitro* (Goryshin and Reznikoff, 1998; Reznikoff et al., 2004; Mutaqin et al., 2011). One method involves making a single PCR product or a synthetic DNA fragment for direct introduction into the genome. The other involves combining a PCR product or a synthetic gene with a vector that provides all of the elements required for introduction and expression of the inserted gene. We have successfully used these methods to establish that deletion of the *nusG* gene encoding a protein associated with RNA polymerase results in the observed loss-of-fitness phenotype.

## Materials and methods

### Bacterial strains and growth conditions

*Mycoplasma mycoides* strains JCVI-syn1.0 (Gibson et al., 2010) and JCVI-syn1.0 ΔL (Suzuki et al., in preparation) were grown in SP-4 liquid medium (Karas et al., 2014) or SP-4 solid medium (containing 1% agar and 150 mg/L X-gal). ΔL denotes the replacement of the genes MMSYN1_0840 (*nusG*) - MMSYN1_0846 with a deletion cassette containing the *KanMX4* marker (Wach et al., 1994). For marker-driven complementation, the solid medium also contained 10 mg/L puromycin.

### Preparation of transposomes

For the marker-less approach, a 1700-bp fragment was amplified using PrimeSTAR GXL DNA Polymerase (Takara). This fragment included a 1642-bp genomic region flanked by 19-bp mosaic ends (Epicenter), as well as 5 base pairs to create a PshA1 restriction site and 5 bp of random bases to enable PshAI cleavage at either end (Supplementary Figure 1). The primers are described below. The PCR product was purified using Qiagen PCR Purification Kit and digested with PshA1 restriction enzyme (New England Biolabs). The digested fragment was purified using extraction with phenol, chloroform and isoamyl alcohol (Sigma) and concentrated using ethanol precipitation. The concentration of DNA was adjusted to 100 ng/μL in Tris EDTA (TE) buffer. 2 μL of the digested and purified fragment was mixed with 2 μL of 100% glycerol and 4 μL of EZ-Tn5 Transposase (from EZ-Tn5 <TET-1> Insertion Kit, Epicenter) and incubated for 30 min at room temperature to prepare Tn5 transposomes. 4 μL of the resulting mixture was used for mycoplasma transformation (see below).

For the marker-driven approach, the pLS-Tn5-Puro vector was constructed first. For this construction, a *Ptuf::pac* cassette was amplified from the pRK78 plasmid (gift from R. Krishnakumar) using the Tn-puro-F and Tn-puro-R primers (see Primers below), as well as Phusion High-Fidelity PCR Master Mix (New England Biolabs). This PCR product was used as a template in a PCR reaction with the primers Ter-puro-F and Ter-puro-R to attach terminator sequences to the ends of the *Ptuf::pac* cassette (Supplementary Figure 2). The resulting fragment was cloned into the SmaI cloning site of the EZ-Tn5 pMOD2 <MCS> vector (Epicenter) to produce the pLS-Tn5-Puro vector.

A fragment containing the open reading frame (ORF) of *nusG* (642 bp), as well as flanking 40-bp sequences with homology to the pLS-Tn5-Puro vector, was generated using PCR with the primers described below and genomic DNA of *M. mycoides* JCVI-syn1.0 as a template (Supplementary Figure 3). A linear fragment containing the pLS-Tn5-Puro vector (3350 bp) was prepared using PCR with primers listed below. Gibson assembly (Gibson et al., 2009) was then performed to combine the insert and the vector. The products from the assembly were introduced into *E. coli* to establish a plasmid. Three out of ten colonies tested had the correct assembly as assayed using digestion with PshA1. Digested DNA samples from these correct colonies were pooled, purified using extraction with phenol, chloroform and isoamyl alcohol and concentrated using ethanol precipitation. The purified fragments adjusted to 100 ng/μL were used to prepare Tn5 transposomes as described above. 4 μL was used for mycoplasma transformation.

For the Tn5 protection experiment, the pLS-Tn5-Puro was digested with PshA1. Digested DNA was purified using extraction with phenol, chloroform and isoamyl alcohol and concentrated using ethanol precipitation. 2 μL of the purified fragments adjusted to 400 ng/μL was combined with 2 μL of 100% glycerol and 4 μL of EZ-Tn5 Transposase. After vortexing and 30-min incubation at room temperature, 4 μL was used for mycoplasma transformation.

### Mycoplasma transformation

Twenty mL of mycoplasma culture at approximately pH 7 was centrifuged at 9000 RCF for 8 min at 10°C in a 50-mL Falcon tube. After the supernatant was decanted, the cells were resuspended in 20 mL of S/T buffer (0.5 M sucrose, 10 mM Tris-HCl; pH = 6.5) and centrifuged as before. The supernatant was decanted, and the residual solution was removed using a 1-mL pipette tip. The pellet was then gently resuspended in 750 μL of 0.1 M CaCl_2_ and incubated for 30 min on ice. 250 μL of the solution containing the prepared cells was transferred to a 50-mL Falcon tube containing 4 μL of the transposome solution. The cells and the transposomes were mixed by triturating a few times using a 1-mL tip. 2 mL of 70% PEG_6000_ (polyethylene glycol; Sigma-Aldrich; cat. no. 25322-68-3) dissolved in S/T buffer (see following paragraph for preparation) was added to the tube, and the transformation mixture was triturated a few times using a 10-mL pipette and incubated at room temperature for 2 min. After the incubation, 20 mL of S/T buffer was added to the cells. The cells were dispersed by inverting the tube 5–7 times and centrifuged at 10,000 RCF at 10°C for 15 min. The supernatant was removed first by decanting and then by pipetting out the residual solution using a 1-mL tip. The pellet was not visible, but was present. The cells were resuspended in 15 mL (marker-driven approach) or 20 mL (marker-less approach) of SP4 medium using a 10-mL pipet and incubated for six hours (marker-driven approach) or 24 h (marker-less approach) at 37°C. In the marker-driven approach, 500 μL of the culture was plated on SP-4 agar medium containing 10 mg/L puromycin and incubated at 37°C. Colonies appeared in 2–4 days. In the marker-less approach, the culture was passaged as described in **Figure 4** below.

Because 70% PEG_6000_ is solid at room temperature, we first dissolved it by heating in a water bath at 50°C for a few hours with thorough mixing by inverting. After this, 35 mL of the solution was added to a fresh 50-mL Falcon tube which was placed in a 250-mL beaker filled with 37°C water. This was allowed to stand at room temperature for 15 min prior to addition to the sample containing cells and transposome.

### PCR analysis of mycoplasma transformants

Fast-growing mycoplasma cells from complementation experiments were analyzed for the presence of the complementation cassettes and the deletion cassette for the L gene cluster using primers listed below. Qiagen Multiplex PCR Kit was used for the non-multiplex PCR. 1 μL of the mycoplasma culture was directly introduced as a template into a 20-μL PCR reaction.

### Primers

The following primers were used in this study.

Assembly of the pLS-Tn5-Puro vector

Tn-puro-F ctgtctcttatacacatctcaaccatcatcgatgaattttctcgggtgttctcgcatattggctctattttttgaattaagtattaaata

Tn-puro-R ctgtctcttatacacatctcaaccctgaagctcttgttggctagtgcgtagtcgttggcttaagcaccaggttttctagtcatacacca

Ter-puro-F tttaataataaaaaatcgggatttcccgatttttttgtattttttgaattaagtattaaa

Ter-puro-R tttaataataaaaaatcgggatttcccgatttttttgttaagcaccaggttttctagtc

Amplification of the complementation fragment for the marker-less approach

Forward primer gcatcgacagctgtctcttatacacatctttttttaattaaaataaatacatatataata

Reverse primer attaagacagctgtctcttatacacatctatttaatactccttaaacaatatttttatgt

Amplification of the insert for the marker-driven approach

Forward primer tcctagaacttggtgtatgactagaaaacctggtgcttaaatgacttatgaagaaatcaa

Reverse primer ccctttaataataaaaaatcgggatttcccgatttttttgttaatattctttaataggtt

Amplification of pLS-Tn5-Puro for the marker-driven approach

Forward primer caaaaaaatcgggaaatcccgattttttattattaaaggggatcctctagagtcgacctg

Reverse primer ttaagcaccaggttttctagtcatacaccaagttctaggaccttcaggaacttcaacatc

Confirmation of the left deletion junction in JCVI-syn1.0 ΔL (Supplementary Figure 4)

TEFterm_seq atatggtattgataatcctg

MMSYN1_0840_GTOF tgaactggatcaggaagtttagg

Confirmation of the right deletion junction in JCVI-syn1.0 ΔL (Supplementary Figure 4)

TEFpr_seq atgcaaatgattatacatgg

MMSYN1_0846_GTOR ggattgtctcctgtacaattcaga

### Calculation of growth rates

Growth rates were determined by quantifying the increase in cell-associated nucleic acid in logarithmic-phase liquid cultures grown statically in SP-4 medium at 37°C (to be published elsewhere). Briefly, overnight cultures were started from serially diluted cell suspensions. A suitable culture was selected in the morning, diluted and aliquoted into replicate 1.5-mL tubes. At selected times, tubes were removed and transferred to ice to arrest growth. After final collection, each sample (0.80 mL) was underlain with a 0.40-mL sucrose cushion (0.5 M sucrose, 20 mM Tris-HCl; pH 7.5). Cells were sedimented by centrifugation at 16,000 RCF at room temperature for 10 min. Medium (with proteins that interfere with the PicoGreen assay) was aspirated, and the remaining clear cushion was adjusted to 100 μL. Cells were lysed by trituration after adding sodium dodecyl sulfate (SDS) to a final concentration of 0.1 % (w/v) in TE buffer in a final volume of 150 μL, and the lysate was then diluted to 0.01% SDS with TE. To quantify dsDNA (with a lesser component of cellular dsRNA), equal volumes (80 μL) of each lysate and Quant-iT PicoGreen reagent (prepared as described by the manufacturer; Molecular Probes, Life Technologies) were mixed in individual wells of opaque black 96-well plates (Costar; cat. no. 3915) and incubated at room temperature for 5 min. Fluorescence was measured using a FlexStation 3 fluorimeter (Molecular Devices) with excitation at 488 nm, emission collected at 525 nm, and a cutoff setting of 515 nm. Relative fluorescence units (RFU) were plotted as log_2_(RFU) vs. time (in minutes) and the doubling times were calculated from the slopes of exponential regression curves (*R*^2^ values shown in the legend for **Figure 5** below) using the formula: doubling time = ln2/exponential rate.

## Results

### The marker-less approach to complementation

We explored the ability to restore one or a few genes in a deletion mutant mycoplasma strain without using whole genome construction, in order to accelerate the characterization of gene functions and the optimization of gene contents for engineering strains for various purposes. To establish a method for directly introducing genes into mycoplasmas that is simple and flexible enough to accommodate assorted sequence features possibly included within the genes of interest, we applied *in vitro* reconstitution of active transposon complexes (Goryshin and Reznikoff, 1998; Reznikoff et al., 2004) using DNA fragments generated *in vitro* (for example, PCR fragments and synthetic DNA fragments).

**Figure 2:**
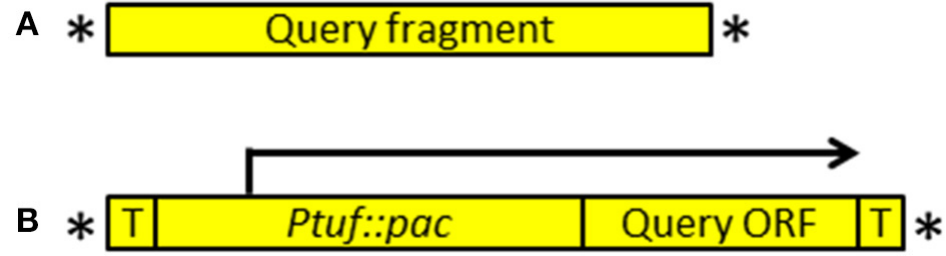
**Designer transposons used in a marker-less approach (A) and a marker-driven approach (B)**. Asterisk denotes a 19-bp mosaic end recognized by Tn5 transposon. In **(A)**, a query fragment can be generated using polymerase chain reaction or prepared as a synthetic DNA fragment. In **(B)**, a query ORF is cloned downstream of the *pac* puromycin resistance marker. The start codon of the query ORF immediately follows the stop codon of the *pac* gene (see Supplementary Figure 2). This ORF can be amplified from a template or prepared as a synthetic DNA block. The *tuf* promoter that drives the expression of the *pac* gene also drives the expression of the query ORF. T denotes transcriptional terminator.

Among the seven genes deleted in a slow-growing strain of the synthetic cell, JCVI-syn1.0 ΔL (Figure 1), we considered restoring the *nusG* gene because it had the smallest number of transposon insertions in a transposon bombardment study (Suzuki et al., in preparation). The intergenic sequence between the *nusG* ORF and the upstream gene is only 16 bp long and may not contain a promoter for *nusG*. Therefore, we amplified a contiguous sequence from the original JCVI-syn1.0 genome containing the *nusG* gene, along with the upstream gene and its 5′ flanking region to generate a PCR fragment (the “query fragment” shown in Figure 2). Within the primers for amplification, we included the 19-bp mosaic ends and interlaced PshAI sites for exposing the necessary 5′-phosphate at the mosaic ends for transposase loading (see Materials and Methods). After loading the Tn5 transposase onto the fragment, the reaction mixture was used to transform the deletion strain JCVI-syn1.0 ΔL.

**Figure 3:**
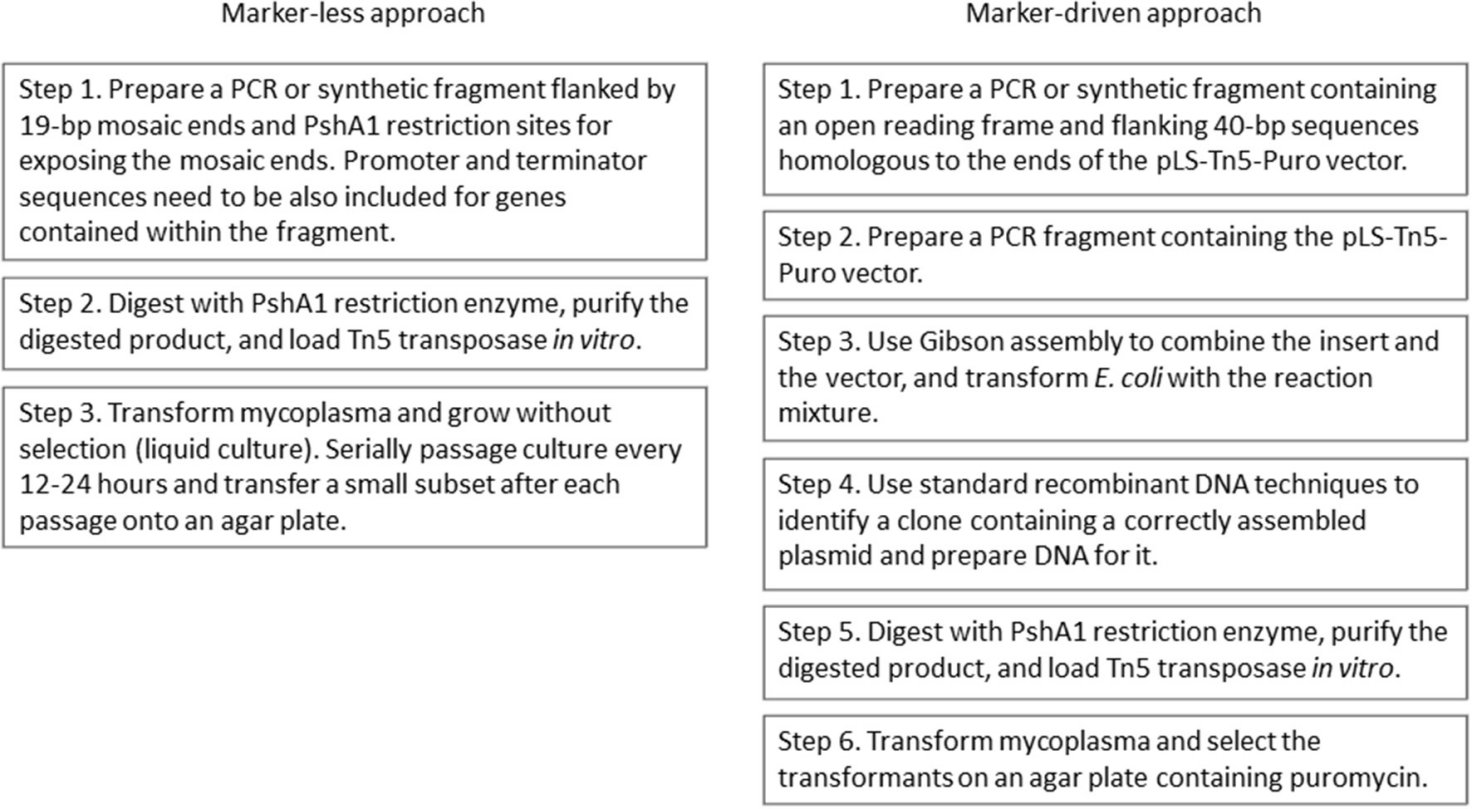
**Flow charts of procedures for complementation of mycoplasma mutants using the marker-less approach and the marker-driven approach**.

**Figure 4:**
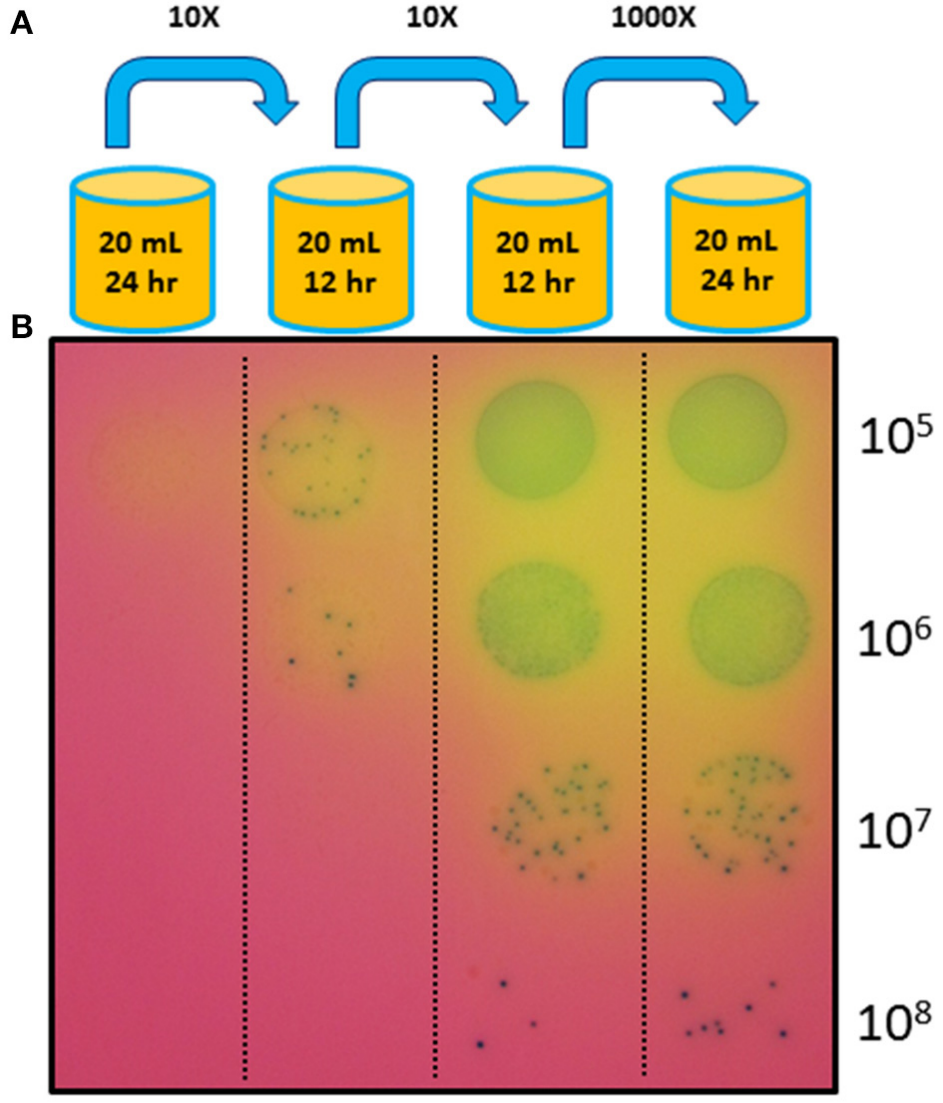
**Complementation using the marker-less approach**. **(A)** Transformed mycoplasma cells were grown without any antibiotic selection in liquid culture through three serial passages with the dilution levels and culture durations indicated. After a new culture was started, the previous culture was stored at 4°C. **(B)** After the fourth culture, cells from each of the four successive cultures were diluted and plated on agar medium (also not containing any antibiotic) with X-gal to visualize the colonies. The enrichment of fast-growing colonies in later cultures suggests that the transformed and complemented cells overtook the population during later cultures.

Based on the expectation that transformants with a growth advantage conferred by the introduced *nusG* fragment would outcompete the untransformed JCVI-syn1.0 ΔL cells, we cultured the transformation mixture without any marker selection, relying on selection for fast growers during a regimen of cell growth over several generations (Figure 3). We found that within two passages, representing roughly 100-fold amplification of the initial transformation culture, fast-growing cells (putative transformants) became the majority of the population (Figure 4).

To confirm that the fast-growing cells are transformants, we isolated colonies and analyzed the genotypes using PCR (Supplementary Figure 4). We found that five out of five colonies contained the introduced *nusG* fragment. All of the colonies also contained the deletion cassette used to replace the L gene cluster within the genome, as assayed using PCR primers amplifying the junctions between the deletion cassette and the relevant genomic sequence. This result demonstrates the feasibility of marker-less transformation for rescuing a slow-growing mutant of synthetic *M. mycoides*.

**Figure 5:**
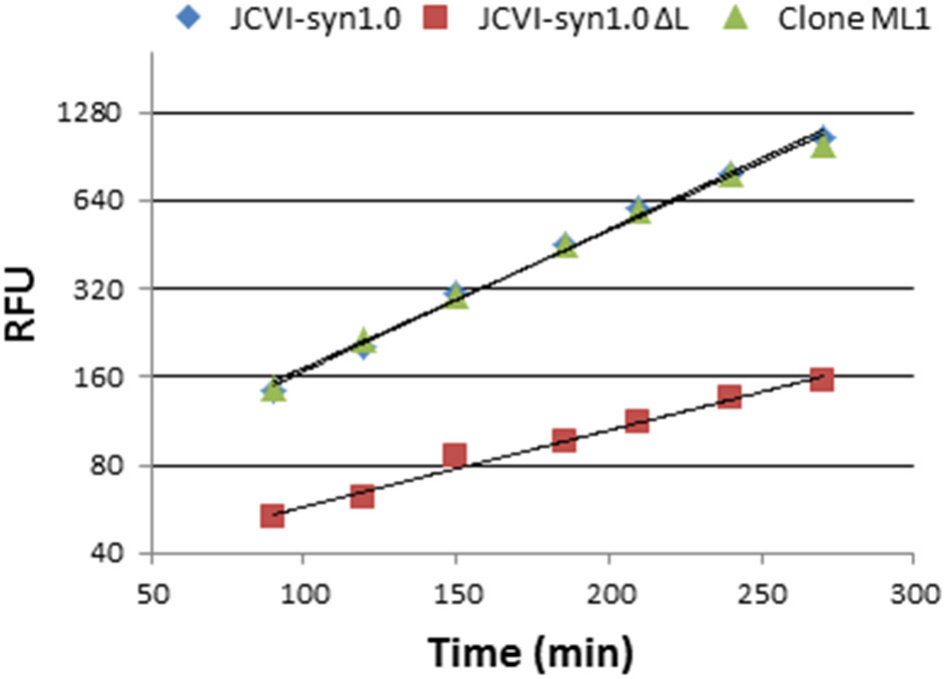
**Restoration of the replication rate of JCVI-syn1.0 ΔL by inserting complementation fragment 1 (containing *nusG*) via the marker-less approach**. The line for JCVI-syn1.0 (blue) and the line for rescued clone ML1 (green) overlap. Doubling times calculated from PicoGreen fluorescence (RFU) are 62 min for JCVI-syn1.0 (*y* = 54.732e^0.0112x^, *R*^2^ = 0.9951), 64 min for complemented clone ML1 (*y* = 58.374e^0.0108x^, *R*^2^ = 0.9951), and 116 min for JCVI-syn1.0 ΔL (*y* = 31.425e^0.006x^, *R*^2^ = 0.983).

To examine the growth rate of the rescued mutant in detail, we used a PicoGreen method to measure the increase in DNA content during culture (see Materials and Methods). DNA content is expected to be a more direct indicator of cell number than culture pH or redox activity. Unlike measurement of colony forming unit or optical density, this method is not affected by variable cell aggregation that may happen during mycoplasma cultures. From the linear portion of a logarithmic plot, we calculated the growth rates (Figure 5). The respective doubling times were 62 min for JCVI-syn1.0, 116 min for JCVI-syn1.0 ΔL, and 64 min for the complemented clone ML1, indicating that the introduced fragment completely restored the mutant growth rate to normal.

### The marker-driven approach to complementation

Because two genes were introduced into the deletion mutant in the experiment described above, it is formally possible that the upstream gene rather than *nusG* was responsible for rescuing the growth defect of the strain. The most straightforward explanation for the rescue by the upstream gene would be that the upstream gene is not deleted, but still is perturbed by the deletion of the neighboring gene cluster (cluster L), that this perturbation results in slow growth and that the activity of the upstream gene is restored by the introduction of the fragment containing the two genes.

To clarify the result from the previous experiment and concurrently to establish a more general tool for complementation, we generated a vector enabling facile expression of any ORF in *M. mycoides*. This vector contains the *tuf* promoter driving the *pac* puromycin resistance gene and the 19-bp mosaic ends interlaced with the PshAI restriction sites for exposing the mosaic ends. To efficiently express the introduced ORF, a promoter, ribosomal binding site and a transcriptional terminator would need to be also included. Because the transposon insert size was known to inversely correlate with transposon efficiency (consistent data are presented in Table 1), we took a marker-driven strategy where the introduced ORF is joined with the puromycin marker so that the promoter of this marker drives both of these elements.

**Table 1:** **Colony counts from complementation of JCVI-syn1.0 ΔL using the marker-driven approach**.

**Mycoplasma strain**	**Transposon content and size**	**Number of colonies (observed morphology)**
JCVI-syn1.0 ΔL	None	0
JCVI-syn1.0 ΔL	Vector only (puroR) 986 bp	4575 (small colony size)
JCVI-syn1.0 ΔL	Vector and *nusG* (puroR) 1628 bp	2000 (large colony size)

*One 30th of the transformation mixture was plated*.

An interesting feature of at least some mycoplasma genomes is that numerous annotated genes have little or no sequence separating them from their adjacent upstream genes. Often there is no discernible sequence for a ribosomal binding site for such genes. Moreover, while Shine-Dalgarno sequences may be embedded in the upstream ORF, an alternative mechanism may exist for translating leader-less transcripts in mycoplasmas (Weiner et al., 2000). Based on the organization of many genes in mycoplasma genomes, our design included the placement of an ORF directly after the stop codon of the *pac* puromycin resistance gene.

**Figure 6:**
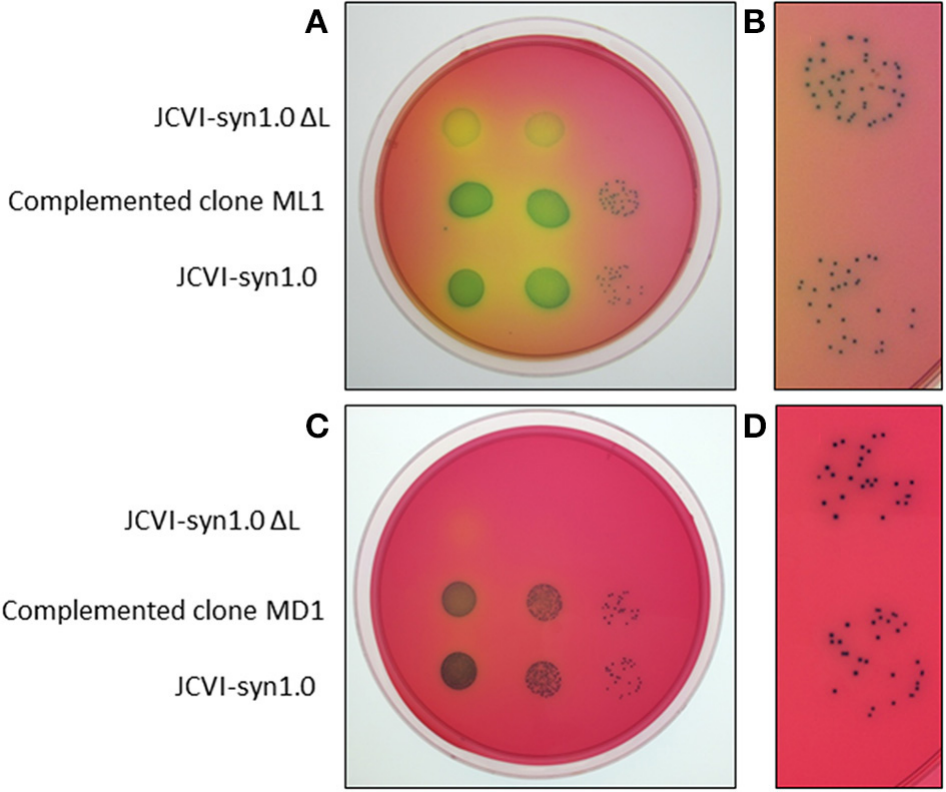
**Comparison of a rescued JCVI-syn1.0 ΔL clone with the positive- and negative-control strains for a clone (ML1) obtained using the marker-less approach (A,B) and for a clone (MD1) obtained using the marker-driven approach (C,D)**. **(B)** and **(D)** are magnified views of colonies in **(A)** and **(C)**, respectively, showing rescued mutant **(top)** and wild-type JCVI-syn1.0 **(bottom)** populations.

**Figure 7:**
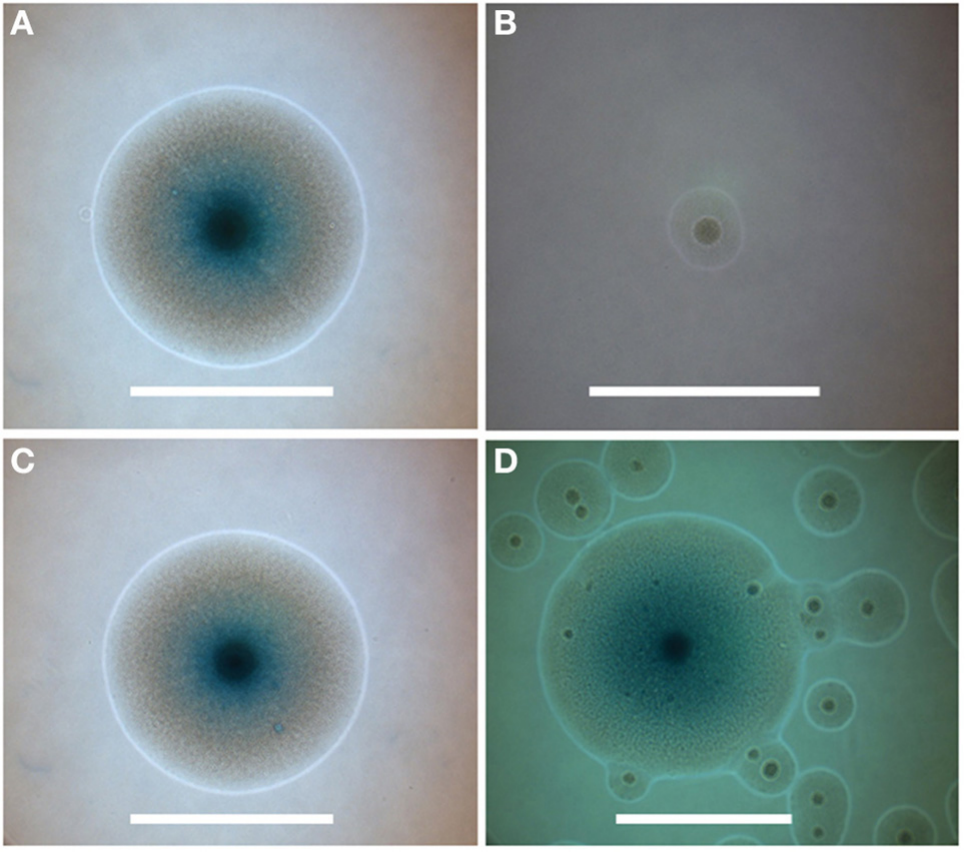
**Colony phenotypes associated with *nusG* deletion and rescue**. **(A–C)** Individual colonies were photographed 72 h after plating on agar medium from sparse fields where colony growth would be unaffected by neighboring colonies. Colonies represent **(A)** JCVI-syn1.0, **(B)** JCVI-syn1.0 ΔL, and **(C)** complemented clone ML1. **(D)** represents a field of colonies from the second culture in Figure 4, showing the initial emergence of the faster growing colonies among the more prevalent smaller colonies within the population derived from transformation of JCVI-syn1.0 ΔL using the marker-less approach. Centers of colonies display the typical mycoplasma “fried-egg” characteristic. Blue color is from expression of *lacZ* in medium containing X-gal (smaller colonies had not yet developed color at the time of assay). Bars represent 500 μm.

Using Gibson assembly (Gibson et al., 2009), we introduced the ORF of the *nusG* gene into the developed vector. Transformation of the deletion strain using the active transposon complex generated from this construct resulted in numerous puromycin-resistant colonies (Table 1). In addition, we noticed that the sizes of the colonies were large when the *nusG* construct was used, whereas the puromycin-resistant colonies were small when the vector alone was used. We then found that six out of six colonies obtained with the *nusG* construct were correct transformants based on the presence of the introduced *nusG* fragment and the deletion cassette in the genome. When a transformant was compared to the JCVI-syn1.0 strain lacking the L deletion, colony sizes were indistinguishable (Figures 6, 7). This result confirmed that the *nusG* is required for normal growth of the synthetic mycoplasma cell. Moreover, a facile tool for rapidly testing the functions of ORFs in synthetic mycoplasma is demonstrated.

### Transposase-dependent protection of the introduced fragment from restriction

Because the JCVI-syn1.0 ΔL strain contained all six restriction systems, it is striking that our small scale transformation (for example, with 200 ng of DNA) resulted in numerous colonies. Our constructs contained sequences that would be cleaved by the restriction enzymes of these systems. To investigate the possibility that the transposase loaded onto our DNA material contributed to the attenuation of restriction, we compared the transformation efficiencies between the JCVI-syn1.0 strain with the six restriction systems and a strain, JCVI-syn1.0 Δ1-6 (Karas et al., 2013), that lacks all of them. When a vector transposon containing only the puromycin marker was combined with the transposase and introduced into these strains, the transformation efficiency with the restriction-capable strain was roughly half of that with the restriction-defective strain (Table 2). In contrast, when a naked plasmid with an ability to propagate in *M. mycoides* (to be published elsewhere) was used, the number of colonies was reduced by more than 50-fold due to restriction (Table 2). Attenuation of restriction may be due to rapid integration of the introduced DNA into the genome via transposition or to transposase-bound DNA somehow being a poor substrate for restriction enzymes. Additional experiments are needed to clarify the mechanism for protection. Nevertheless, the option to introduce DNA fragments into strains still containing restriction systems is valuable for broadening the spectrum of strains that can be engineered using our transposon-mediated methods (for example, to include non-laboratory strains).

**Table 2:** **Protection of transposase-loaded DNA from restriction**.

**Experiment**	**Mycoplasma strain**	**pH of culture at harvest**	**Number of mycoplasma colonies after transformation**		
			**Tn5 designer transposome (puromycin)**	**Plasmid**	**No DNA**
1	JCVI-syn1.0	6.6	117,000	7350	0
	JCVI-syn1.0 Δ1-6	6.8	222,450	696,000	0
2	JCVI-syn1.0	6.7	66,960	3660	0
	JCVI-syn1.0 Δ1-6	6.7	106,980	223,980	0

*Four hundred nanograms of DNA were used for transposome samples. Four hundred and one hundred nanograms were used in experiments 1 and 2, respectively, for plasmid samples. Colony numbers normalized to represent the whole transformation mixtures are shown. The pH values of the cultures used for transformation procedures are shown. pH of the medium decreases as mycoplasma cells grow, and the pH range of 6.6–6.8 is roughly associated with late log phase*.

## Discussion

Using two new methods for introducing short DNA molecules into *M. mycoides*, we have shown that the *nusG* gene encoding a protein that interacts with RNA polymerase is required for normal growth rate in this organism. In *E. coli*, the *nusG* product is known to have multiple functions related to transcription such as enhancement of elongation, termination, and silencing of foreign DNA (Cardinale et al., 2008; Tomar and Artsimovitch, 2013). Future efforts are needed to identify the specific *nusG* defects that resulted in the slow growth phenotype in *M. mycoides*.

Our first method can be used to directly convert a PCR fragment or a synthetic DNA fragment containing almost any sequence into an active transposon complex for introduction into *M. mycoides*. Our second method is also straightforward, in that a DNA sequence representing any ORF can be rapidly introduced into a vector to facilitate the testing of the ORF in *M. mycoides*. To our knowledge, this is the first published study using a system for *in vitro* loading of transposase in mycoplasmas for complementation tests. Moreover, we have found that transposase-bound DNA is protected from restriction in our study.

We have observed in the marker-driven approach that the ORF of *nusG* can directly follow the puromycin marker gene in a construct for conferring full complementing function. There is no discernible ribosomal binding site near the start codon of the *nusG* gene. This design may be effective only in mycoplasmas where numerous genes in the genomes appear to lack intergenic sequences. However, it is possible that more robust expression will be generally achieved with the inclusion of a ribosomal binding site. Therefore, users of this technology may consider including a ribosomal binding site in a design for a PCR or synthetic fragment.

As more deletion mutants with fitness defects are encountered in the study of synthetic *M. mycoides*, we expect the demand for the developed methods to grow. Based on the ease of each technique, the throughput should be sufficiently high to enable a genome-wide scan of genomic fragments or specific ORFs for complementing functions. With the applicability to synthetic DNA fragments, the value of our approaches is expected to further expand as genetic elements are better defined to enable designing of synthetic genes with novel and precisely predictable functions. The same approaches are likely to work in mycoplasmas other than the synthetic cell and also in other bacteria. Therefore, the facile tools developed in this study may be applied to advance systems biology studies in numerous organisms.

### Conflict of interest statement

The authors declare that the research was conducted in the absence of any commercial or financial relationships that could be construed as a potential conflict of interest.
