# Supplementary material for: Rescue of mutant fitness defects using in vitro reconstituted designer transposons in Mycoplasma mycoides
Source: Front Microbiol. 2014 Jul 23;5:369. doi: 10.3389/fmicb.2014.00369 (PMC4107850; doi:10.3389/fmicb.2014.00369)
Supplement: Supplementary file 1 [file Presentation1.PDF]

Supplementary Material

Rescue of mutant fitness defects using *in vitro* reconstituted designer transposons in *Mycoplasma mycoides*

Bogumil J. Karas[1]*, Kim S. Wise[1,2], Lijie Sun[1], J. Craig Venter[1,3], John I. Glass[1,3], Clyde A. Hutchison III[1], Hamilton O. Smith[1], Yo Suzuki[1]*

[1]Department of Synthetic Biology and Bioenergy, J. Craig Venter Institute, La Jolla, California, USA
[2]Department of Molecular Microbiology and Immunology, University of Missouri, Columbia, Missouri, USA
[3]Department of Synthetic Biology and Bioenergy, J. Craig Venter Institute, Rockville, Maryland, USA

*** Correspondence:**

Bogumil J. Karas, Department of Synthetic Biology and Bioenergy, J. Craig Venter Institute, 4120 Capricorn Lane, La Jolla, California, 92037, USA.
bkaras@jcvi.org
Yo Suzuki, Department of Synthetic Biology and Bioenergy, J. Craig Venter Institute, 4120 Capricorn Lane, La Jolla, California, 92037, USA.
ysuzuki@jcvi.org

1. Supplementary Figures

Forward primer
5'-gcatc**GAC**AGCT**GTC**TCTTATACACATCTTTTTTTAATTAAAATAAATACATATATAATA-3'

Reverse primer
5'-attaa**GAC**AGCT**GTC**TCTTATACACATCTATTTAATACTCCTTAAACAATATTTTTATGT-3'

Supplementary Figure 1. PCR primers for constructing a designer transposon for the marker-less approach. The PshAI site (GACNNNNGTC) is in bold. The 19-bp mosaic end is in red. The sequence for amplifying the *nusG* region is underlined. This underlined sequence needs to be adjusted for a specific region of interest. For example, the amplified fragment can contain one or more genes with any number of promoters, ribosomal binding sites, and transcriptional terminators.

tttaataataaaaaatcgggatttcccgatttttttgtatttttgaattaagtattaaataagtgtaaaatatataatagt
aaaaacgccccaaaaggggcagacaaaatagtagaaatatatctattattcttgatttgttatagaaattttaaggaga
aaaaaacatgactgaatataaacctactgttagattagctactagagatgatgttcctagagctgttagaactttagctg
ctgcttttgctgattatcctgctactagacatactgttgatcctgatagacatattgaaagagttactgaattacaagaatt
atttttaactagagttggtttagatattggtaaagtttgggttgctgatgatggtgctgctgttgctgtttggactactcctg
aaagtgttgaagctggtgctgtttttgctgaaattggtcctagaatggctgaattaagtggtagtagattagctgctcaa
caacaaatggaaggtttattagctcctcatagacctaaagaacctgcttggtttttagctactgttggtgttagtcctgat
catcaaggtaaaggtttaggtagtgctgttgttttacctggtgttgaagctgctgaaagagctggtgttcctgctttttttag
aaactagtgctcctagaaatttaccttttttatgaaagattaggttttactgttactgctgatgttgaagttcctgaaggtcc
tagaacttggtgtatgactagaaaacctggtgcttaacaaaaaaatcgggaaatcccgatttttattattaaa

Supplementary Figure 2. Sequence of a *Ptuf::pac* cassette. This cassette was cloned into the sole SmaI site of the EZ-Tn5 PMOD-2 <MCS> transposon construction vector. Sequences containing a *Mycoplasma mycoides* terminator are in red. These were taken from the genome sequence of *M. mycoides* JCVI-syn1.0 (GenBank accession number: CP002027.1, coordinates 9,947-9,983). The *tuf* promoter is underlined. The *pac* puromycin resistance gene is in blue.

Forward primer for amplifying the vector
5'-CAAAAAAATCGGGAAATCCCGATTTTTTATTATTAAAGGGGATCCTCTAGAGTCGACCTG-3'
Reverse primer for amplifying the vector
5'-**TTA**AGCACCAGGTTTTCTAGTCATACACCAAGTTCTAGGACCTTCAGGAACTTCAACATC-3'

Forward primer for amplifying the ORF of interest
5'-TCCTAGAACTTGGTGTATGACTAGAAAACCTGGTGCT**TAAATG**ACTTATGAAGAAATCAA-3'
Reverse primer for amplifying the ORF of interest
5'-CCCTTTAATAATAAAAAATCGGGATTTCCCGATTTTTTTGTTAATATTCTTTAATAGGTT-3'

Supplementary Figure 3. Primers for constructing two PCR fragments (vector and insert) to be assembled to make a plasmid for the marker-driven approach. The vector fragment based on pLS-Tn5-Puro vector is constant for any query open reading frame (ORF). Each of the primers for amplifying the query ORF includes a 40-bp sequence homologous to a vector end (for example, the 40-bp sequence in the forward primer of the query is homologous to the "reverse" end of the vector). The stop codon for the puromycin resistance gene is indicated in red. The start codon for the query open reading frame is in green. 20-bp sequences for amplifying the ORF (*nusG* in this case) are underlined. These sequences can be altered depending on the ORF to be introduced into the construct.

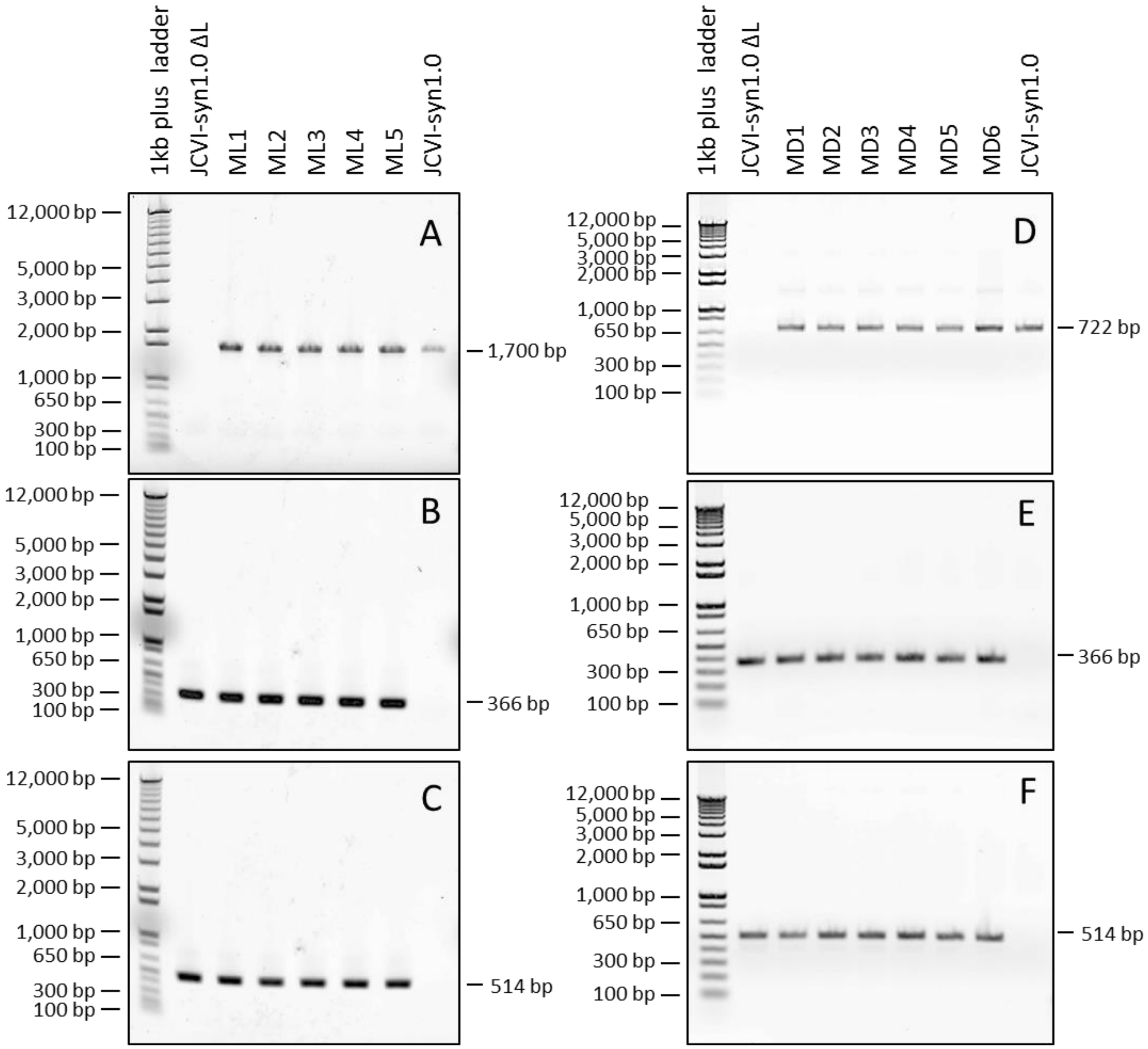

Supplementary Figure 4. PCR confirmation of the genotypes of complemented JCVI-syn1.0 ΔL clones for the marker-less approach (A-C; ML clones) and the marker-driven approach (D-F; MD clones). (A, D) The original forward and reverse primers for constructing the complementation fragments for the marker-less approach (A) and the marker-driven approach (D) were used. (B, E) Primers designed to amplify the left deletion junction were used. Genes MMSYN1_0840-MMSYN1_0846 were replaced with a deletion cassette in JCVI-syn1.0 ΔL. One primer annealed to a sequence outside of this gene cluster, and the other primer annealed to the deletion cassette. (C, F) Primers designed to amplify the right deletion junction were used. The sizes of some of the bands in the 1-kb plus ladder are indicated on the left side of each gel. The expected size of each PCR product is indicated on the right side.
